# 3D Hepatic Cultures Simultaneously Maintain Primary Hepatocyte and Liver Sinusoidal Endothelial Cell Phenotypes

**DOI:** 10.1371/journal.pone.0015456

**Published:** 2010-11-12

**Authors:** Yeonhee Kim, Padmavathy Rajagopalan

**Affiliations:** 1 Department of Chemical Engineering, Virginia Polytechnic Institute and State University, Blacksburg, Virginia, United States of America; 2 ICTAS Center for Systems Biology of Engineered Tissues, Virginia Polytechnic Institute and State University, Blacksburg, Virginia, United States of America; Université de Technologie de Compiègne, France

## Abstract

Developing *in vitro* engineered hepatic tissues that exhibit stable phenotype is a major challenge in the field of hepatic tissue engineering. However, the rapid dedifferentiation of hepatic parenchymal (hepatocytes) and non-parenchymal (liver sinusoidal endothelial, LSEC) cell types when removed from their natural environment *in vivo* remains a major obstacle. The primary goal of this study was to demonstrate that hepatic cells cultured in layered architectures could preserve or potentially enhance liver-specific behavior of both cell types. Primary rat hepatocytes and rat LSECs (rLSECs) were cultured in a layered three-dimensional (3D) configuration. The cell layers were separated by a chitosan-hyaluronic acid polyelectrolyte multilayer (PEM), which served to mimic the Space of Disse. Hepatocytes and rLSECs exhibited several key phenotypic characteristics over a twelve day culture period. Immunostaining for the sinusoidal endothelial 1 antibody (SE-1) demonstrated that rLSECs cultured in the 3D hepatic model maintained this unique feature over twelve days. In contrast, rLSECs cultured in monolayers lost their phenotype within three days. The unique stratified structure of the 3D culture resulted in enhanced heterotypic cell-cell interactions, which led to improvements in hepatocyte functions. Albumin production increased three to six fold in the rLSEC-PEM-Hepatocyte cultures. Only rLSEC-PEM-Hepatocyte cultures exhibited increasing CYP1A1/2 and CYP3A activity. Well-defined bile canaliculi were observed only in the rLSEC-PEM-Hepatocyte cultures. Together, these data suggest that rLSEC-PEM-Hepatocyte cultures are highly suitable models to monitor the transformation of toxins in the liver and their transport out of this organ. In summary, these results indicate that the layered rLSEC-PEM-hepatocyte model, which recapitulates key features of hepatic sinusoids, is a potentially powerful medium for obtaining comprehensive knowledge on liver metabolism, detoxification and signaling pathways *in vitro*.

## Introduction

The liver is one of the largest organs in our bodies. It performs a multitude of functions such as metabolism, detoxification, and mediation of the body's complex defense mechanisms. Two of the most commonly observed cell types in the liver are hepatocytes and liver sinusoidal endothelial cells (LSECs). Together, they account for more than 80% of the liver's mass. Hepatocytes are responsible for several metabolic and detoxification functions that are unique to the liver [1]. LSECs exhibit characteristics that are distinct from endothelial cells that line other blood vessels. They participate in metabolic activities, exhibit fenestrae [1], [2] and are often the initial target of hepatic toxicants [3]. LSECs also function as a scavenger system in the liver by removing waste macromolecules and play a vital role in the balance of lipids, cholesterol and vitamins [4]–[10].

These primary hepatic cell types are well known to exhibit a dramatic loss in their phenotypic characteristics when removed from the organ [1]. Specifically, hepatocytes and LSECs dedifferentiate within 72h when cultured as monolayers *in vitro* [11]–[14]. Various approaches have been utilized to design *in vitro* hepatocyte cultures in order to maintain their phenotype. For instance, hepatocytes cultured in a collagen sandwich or in 2D co-cultures with non-parenchymal cells remain stable over two an extended period of time [12]–[14], [15]–[21]. More recently, hepatocytes sandwiched between Matrigel layers were reported to exhibit stable function [22]. Despite their advantages, collagen or Matrigel sandwich cultures do not provide the complex multi-cellular environment found *in vivo*, whereas 2D co-cultures do not mimic the layered liver architecture. Maintaining the phenotype of LSECs has proven to be equally difficult. Recently, studies have demonstrated that modulating the microenvironment in LSEC cultures can delay the dedifferentiation of LSECs [23]–[25]. For example, LSECs cultured on extra-cellular matrices derived from the liver, bladder or intestine maintained liver-specific characteristics up to 3 days when cultured individually or up to 7 days when co-cultured with primary hepatocytes [24]. Varying the cellular microenvironment in combination with culturing LSECs with fibroblasts and hepatocytes delayed the dedifferentiation of LSECs up to 14 days [25]. The addition of growth factors such as vascular endothelial cell growth factor (VEGF), hepatocyte growth factor (HGF), and platelet derived growth factor (PDGF) can also prolong the loss of LSEC-specific characteristics [26]–[28]. In spite of these advances, it has proven extremely difficult to *simultaneously* maintain the phenotypes of hepatocytes and LSECs *in vitro*. Consequently, the multitude of critical hepatic functions that hepatocytes and LSECs perform cannot be studied adequately or monitored for long periods *in vitro*.

*In vivo*, hepatocytes and LSECs are separated by a protein-based interface called the Space of Disse [1]. This is an interface comprised of various extracellular matrix proteins and proteoglycans. The ECM is highly organized and composed of proteins (collagen type I, II, IV, and V), glycoproteins (fibronectin, laminin, tenascin, and osteonectin), proteoglycans (heparin sulfate, and chondroitin sulfate), and glycosaminoglycans (hyaluronan). The Space of Disse enables the diffusion of molecules from the fenestrated LSEC layers to the hepatocytes and thereby acts as a “molecular sieve” [1]. We hypothesized that constructing an *in vitro* model that mimicked the Space of Disse might assist in delaying the de-differentiation of hepatic cells. In previous attempts to test this hypothesis, we have reported the assembly of 3D hepatic cellular constructs assembled from primary hepatocytes, human umbilical vein endothelial cells (HUVECs)/human LSECs (hLSECs) and a polyelectrolyte-derived interfacial region that mimics the Space of Disse [29], [30]. We have previously demonstrated that an interface comprised of polyelectrolyte multilayers (PEMs) enables the assembly of the interfacial region with precise control over the height, hydrated thickness and the modulus [29], [30]. Since polyelectrolytes are either cationic or anionic, the presence of a PEM recapitulates the charged environment of the Space of Disse. The PEM was deposited above a layer of hepatocytes and was comprised of cationic (chitosan) and anionic (hyaluronic acid) polyelectrolytes. Since several previous reports have demonstrated the compatibility of chitosan as a substrate to culture hepatocytes, chitosan was selected as the cationic PE [31]–[33]. Hyaluronic acid (HA) is found in the Space of Disse as well as in the basal membranes of connective tissues and is a biomaterial used for the culture of endothelial cells [34], [35]. The height of the PEM was determined to be approximately 30nm and 55nm for five and fifteen layers respectively. The PEM exhibited a high degree of hydration (1.01 mPas) and shear modulus values of approximately 100kPa, similar to those observed within the liver *in vivo* [30]. When human LSECs (hLSECs) were cultured on the PEM-coated hepatocyte layer, the resulting construct exhibited significantly enhanced hepatic properties in comparison to hepatocyte monolayers or collagen sandwich cultures. However, we did not monitor the phenotypes of the LSECs in these earlier studies.

In this study, our goal was to incorporate primary rat LSECs (rLSECs) into the 3D hepatic model to determine if such an arrangement of hepatic cells would prevent or delay the dedifferentiation of both hepatocytes and rLSECs. In the 3D model reported herein, hepatocytes and rLSECs were separated by a nanoscale PEM that could potentially mediate intercellular signaling and ultimately promote heterotypic interactions. The phenotypic characteristics of primary rat hepatocytes and rLSECs were monitored through a combination of hepatocellular-specific assays and immunostaining. Hepatocyte function was monitored through urea and albumin production, cytochrome P450 enzyme kinetics, and by immunostaining for bile canaliculi. For rLSECs, immunostaining for the sinusoidal endothelial marker (SE-1) provided a convenient method to verify their differentiated state.

## Materials and Methods

### Materials

Dulbecco's modified eagle medium (DMEM) containing 4.5 g/L glucose, phosphate-buffered saline (PBS), Earle's balanced salt solution (EBSS), Hank's buffered salt solution (HBSS), ethoxy resorufin, benzyloxy resorufin, resorufin, penicillin, streptomycin, and trypsin-ethylenediaminetetraacetic acid were obtained from Invitrogen Life Technologies (Carlsbad, CA). Type IV collagenase, HEPES (4-[2-hydroxyethyl] piperazine-1-ethanesulfonic acid), glucagon, hydrocortisone, dicumarol, sodium dodecyl sulfate (SDS), hydrogen peroxide, pronase, and glutaraldehye were obtained from Sigma-Aldrich (St. Louis, MO). Endothelial cell growth medium and supplements were obtained from ScienCell Research Laboratories (San Diego, CA). Unless noted otherwise, all other chemicals were obtained and used as received from Fisher Scientific (Pittsburgh, PA).

### Methods

#### Hepatocyte isolation and culture

Primary rat hepatocytes were harvested from female Lewis rats (Harlan, Indianapolis, IN) that weighed between 170–200g. Animal care and surgical procedures were conducted in accordance with procedures approved by Virginia Polytechnic Institute and State University's Institutional Animal Care and Use Committee (IACUC Protocol 10-062-CHE). A two-step *in situ* collagenase perfusion method was utilized to excise the liver [12], [13], [29], [30]. Briefly, rats were anesthetized with 3 L/min of a gas mixture of 3% (v/v) isofluorane/97% oxygen (Veterinary Anesthesia Systems Co., Bend, OR). The liver was perfused with Krebs Ringer Buffer (KRB; 7.13 g/L sodium chloride, 2.1 g/L sodium bicarbonate, 1 g/L glucose, 4.76 g/L HEPES and 0.42 g/L potassium chloride) that contained 1mM EDTA (ethylene diamine tetra acetic acid), followed by perfusion with a 0.1% w/v collagenase (Sigma, Type IV). Cell suspensions were filtered through nylon meshes with porosity ranging from 250 to 62 µm (Small Parts, Inc., Miramar, FL). Hepatocytes were separated using a Percoll (Sigma-Aldrich) density centrifugation technique. Hepatocyte viability was determined by trypan blue exclusion. Hepatocytes were cultured on collagen-coated (rat tail, Type 1 collagen) 6-well sterile tissue culture plates (Becton Dickinson Labware, Franklin Lakes, NJ) and were maintained in culture medium that consisted of DMEM supplemented with 10% heat-inactivated fetal bovine serum (Hyclone, UT), 200 U/mL penicillin, 200 µg/mL streptomycin, 20 ng/mL epidermal growth factor (BD Biosciences, San Jose, CA), 0.5 U/mL insulin (USP, Rockville, MD), 14 ng/mL glucagon and 7.5 µg/mL hydrocortisone. A collagen gelling solution was prepared by mixing 9 parts of Type I collagen (BD Biosciences) solution and 1 part of 10× DMEM. Sterile 6-well tissue culture plates were coated with 0.5 ml of the gelling solution and incubated at 37°C for 1h to promote gel formation. Hepatocytes were seeded at a density of 1 million cells/well. Hepatocyte cultures were maintained at 37°C in a humidified gas mixture of 90% air/10% CO_2_. The culture medium was replaced every 24h and medium samples were stored at −20°C for further analysis.

#### Isolation and culture of primary rat LSECs

Primary rat LSECs (rLSECs) were isolated from the supernatant taken from the centrifugation steps for hepatocyte isolation as well as from hepatic tissue fragments treated with pronase (0.02% (w/v)). The resulting cell fraction was added to 25%/50% Percoll/PBS gradients. Cells located at the interface between 25 and 50% Percoll/PBS were collected for further separation. rLSECs were separated from other non-parenchymal hepatic cells through a differential adhesion step involving incubation on a tissue culture plastic surface at 37°C for 30 min. The non-adherent LSECs were maintained in medium supplemented with 5% (v/v) fetal bovine serum, 1% (v/v) endothelial cell growth supplement, 100 U/mL penicillin, and 100 µg/mL streptomycin at 37°C under a humidified gas mixture of 95% air/5% CO_2_.

#### Assembly of 3D constructs of primary rat hepatic cells

Rat hepatocytes were cultured on collagen-gel coated substrates and allowed to spread up to 72 h to form a confluent monolayer [30]. Upon obtaining a confluent cell layer, a polyelectrolyte multilayer (PEM) was deposited. The PEM was comprised of alternating layers of chitosan (200–300kDa, 0.01% (w/v)) and hyaluronic acid (>1×10^6^ kDa, 0.01% (w/v)). PEMs were assembled on hepatocytes by first depositing a cationic PE on the cell layer followed by the anionic PE. The exposure time for each PE solution was approximately 1–2 minutes. The desired number of layers was obtained through the sequential and alternate deposition of PEs. At the end of the deposition procedure, the samples were rinsed in 1× PBS and subsequently maintained in cell-culture medium at 37°C. Thereafter, a layer of primary rat LSECs was seeded. Typically, 25,000 (denoted as 25K) or 50,000 (denoted as 50K) LSECs were added to each sample and non-adherent cells were removed after 2 h. In order to visually observe the 3D primary hepatic cell constructs, LSECs were labeled with a fluorescent, membrane permeable dye (PKH26 Red Fluorescent Cell Linker Kit; Sigma-Aldrich) prior to seeding. The culture medium was replaced every 24hr and medium samples were collected and stored −20°C until further analysis. Cells were observed and imaged under an inverted Nikon TE-2000 (Nikon) microscope coupled with a Hamamatsu CCD camera.

#### Immunostaining for sinusoidal endothelial -1 (SE-1) LSEC marker

LSECs were fixed in a 2% glutaraldehyde/PBS v/v solution for 30 min at room temperature and subsequently incubated with a 0.1% Triton X-100 solution to permeabilize their membrane. The LSEC samples were placed overnight in a blocking solution (1% BSA/PBS w/v) solution at 4°C. The samples were first exposed to a primary antibody (SE-1, Immuno-Biological Laboratories, Minneapolis, MN) for 2h at 37°C and followed by exposure to a FITC-conjugated secondary antibody (Sigma-Aldrich) for 1 hr at room temperature. Images were acquired on a Zeiss LSM confocal microscope.

#### Urea and albumin production by primary rat hepatocytes

Urea concentration of medium samples was determined via its specific colorimetric reaction with diacetyl monoxime using a commercially available assay kit (BUN Assay kit; Stanbio Laboratory, Boerne, TX) [29], [30]. Albumin concentration of medium samples was analyzed by an enzyme-linked immunosorbent assay (ELISA), in triplicate, utilizing a polyclonal antibody to rat albumin (Cappel Laboratories, Aurora, OH). The absorbance was measured on a SpectraMax M2 microplate reader (Molecular Devices, Sunnyvale, CA). Standard curves were generated using purified rat albumin or urea diluted in culture medium. The data reported were normalized to the DNA content of hepatocytes.

#### Separation of hepatocytes and LSECs in 3D constructs

The 3D constructs comprised of primary rat hepatocytes and LSECs were exposed to SE-1 antibody conjugated Dynabeads® (Invitrogen). SE-1 antibody conjugated Dynabeads were obtained by incubating mouse IgG-tagged Dynabeads® (CELLection™ Pan Mouse IgG Kit; Invitrogen) with the SE-l antibody at room temperature for 20 min [28]. Thereafter the cell suspensions were incubated with SE-1 antibody-tagged Dynabeads® for 20 min at 4°C in an orbital shaker. Dynabead®-bound cells (LSEC fractions) were collected by a magnet (DynaMag™-15; Invitrogen) and the supernatant (hepatocytes) were transferred to a new tube. After separation, the hepatocyte and rLSEC fractions were seeded on a collagen-coated or fibronectin-coated substrate respectively. The number of cells for each fraction was counted using a non-destructive imaging method. Approximately fifty images were taken per sample to determine purity. Analysis of the images based upon the distinctive polygonal and elongated morphology for hepatocytes and rLSECs respectively revealed the purity of cell populations to be greater than 95%.

#### Measurement of DNA content

Hepatocytes were lysed in a 0.1% SDS solution and stored at −20°C until further analysis. For DNA measurements, aliquots of cell suspensions were treated with a fluorescent DNA-binding dye (Hoechst 33258, pentahydrate-bis-benzimide; Invitrogen). The fluorescence intensity was measured using a SpectraMax M2 microplate reader (excitation and emission wavelengths were set at 355 nm and 460 nm respectively) and converted to DNA concentration by comparison to a standard curve for calf thymus DNA (Sigma-Aldrich) with concentrations ranging from 0 to 40 µg/ml.

#### Cytochrome P450 enzyme activity (CYP1A1/2 and CYP3A)

Cytochrome P450-dependent ethoxyresorufin *o*-dealkylase (EROD) or benzyloxyresorufin *o*-dealkylase (BROD) activity was induced by adding 3-methylcholanthrene (3MC, Sigma-Aldrich, 2 µM) or dexamethasone (Sigma-Aldrich, 37.5 µM), respectively, to the hepatocyte cultures 48h prior to conducting measurements. Cytochrome-P450 dependent EROD or BROD detoxification activity was measured using ethoxyresorufin or benzyloxyresorufin as substrate. The incubation mixture contained the appropriate resorufin substrate (5 µM) and 80 µM dicumarol diluted in EBSS (Invitrogen) [29], [30], [36]. Aliquots (100 µL) were taken at 5, 15, 25, and 35 minutes after adding the resorufin mixture, transferred to a 96-well plate and the fluorescence intensity was measured using a SpectraMax M2 microplate reader (excitation and emission wavelengths were set to 530 nm and 580 nm respectively). Fluorescence intensity was converted to values of concentration by comparison to a standard curve for resorufin fluorescence with concentrations ranging from 0 to 1000 nM. The rate of resorufin formation (nM/min) was calculated from the early linear increase in the fluorescence curve, normalized to the DNA content in hepatocytes, and defined as cytochrome P450 isoenzyme activity. The absolute values obtained on day 12 for EROD or BROD activity were divided by the baseline activity on day 4 to obtain values of fold change.

#### Di-peptyl peptidase IV (DPP IV) immunostaining to image bile canaliculi

Hepatocyte monolayers as well as rLSEC-Hepatocyte and rLSEC-PEM-Hepatocyte cultures were fixed in a 2% glutaraldehyde/PBS solution, followed by permeabilization for in a 0.1% Triton X-100 solution. The cultures were incubated overnight at 4°C in a 1% BSA/PBS solution. The samples were incubated with a mouse monoclonal antibody to rat DPP IV (Cell Sciences, Canton, MA) and a secondary FITC-conjugated rabbit anti-mouse IgG antibody (Sigma-Aldrich) and imaged using an inverted Zeiss LSM510 confocal microscope [37], [38].

#### Statistical analysis

All data are reported as mean ± standard deviation (S.D.). *t*-tests were conducted to detect differences in the mean values between day 4 and day 12 for each culture. *p*-values were adjusted for multiple hypothesis testing using the Bonferroni correction. Statistically significant samples at an alpha of 0.05 are denoted with an asterisk (*).

## Results

**Figure 1:**
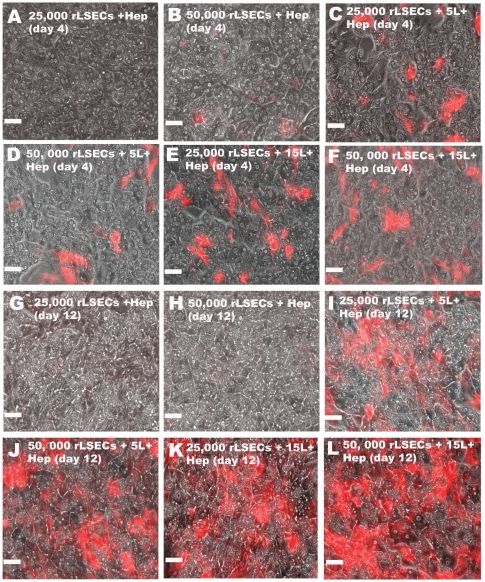
Merged phase-contrast (hepatocytes) and fluorescent images of red-fluorescent rLSECs. Images obtained on day 4 (A–F) and day 12 (G–L) after isolation of hepatocytes and rLSECs. **A**. 25K rLSEC-Hepatocyte, **B**. 50K rLSEC-Hepatocyte, **C**. 25K rLSEC-5L-Hepatocyte, **D**. 50K rLSEC-5L-Hepatocyte, **E**. 25K rLSEC-15L-Hepatocyte, **F**. 50K rLSEC-15L-Hepatocyte. Figures **G–L** represents the same conditions as in **A–F**. Hep = Hepatocytes. Scale bar = 50 microns.

rLSECs were seeded at concentrations of either 5K, 10K, 25K or 50K above PEM-coated primary hepatocytes. In addition, the number of polyelectrolyte layers within the PEM was varied from 5 to 30. Preliminary studies indicated that using 5K and 10K LSECs or PEMs comprised of greater than 15 layers did not maintain or enhance hepatocellular phenotypic functions (*data not shown*). Therefore, we focused our attention on 25K or 50K rLSECs and PEMs comprised of either 5 or 15 layers. In order to determine whether rLSECs would adhere to the underlying PEM, they were incubated with a red-fluorescent non-toxic membrane permeable dye prior to seeding. Images taken over a period of twelve days demonstrated that rLSECs where only adherent when a chitosan-HA PEM was present (**Figure 1**). In the absence of the PEM (denoted as rLSEC-Hepatocyte), rLSECs were not observed 24h post-seeding. Over the culture period, rLSECS proliferated only above the PEM-coated hepatocytes (denoted as rLSEC-PEM-Hepatocyte), indicated by an increase in the area occupied by these cells. These trends prevailed when the PEM was comprised of either 5 or 15 PE layers and at an initial LSEC seeding density of either 25K or 50K (denoted as 25K rLSEC-5L-Hepatocytes, 25K rLSEC-15L-Hepatocytes, 50K rLSEC-5L-Hepatocytes, and 50K rLSEC-15L-Hepatocytes).

**Figure 2:**
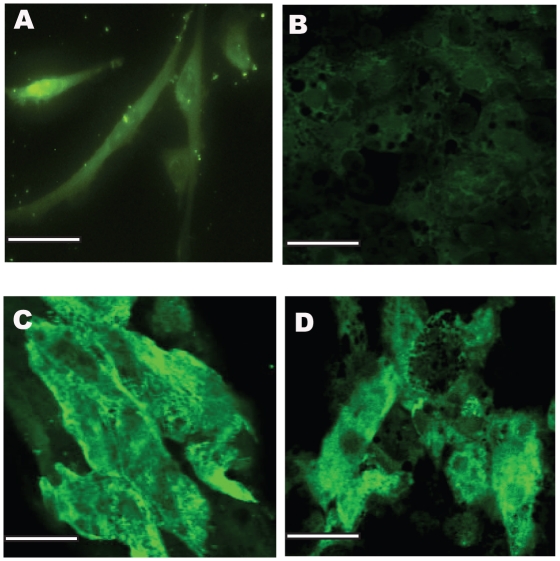
SE-1 immunostaining to monitor rLSEC phenotype, images obtained on day 4 in culture. **A**. rLSEC monolayer, **B**. 50K rLSEC-Hepatocyte, **C**. 50K rLSEC-5L-Hepatocyte, and **D**. 50K rLSEC-15L- Hepatocyte. Scale bar = 50 microns.

**Figure 3:**
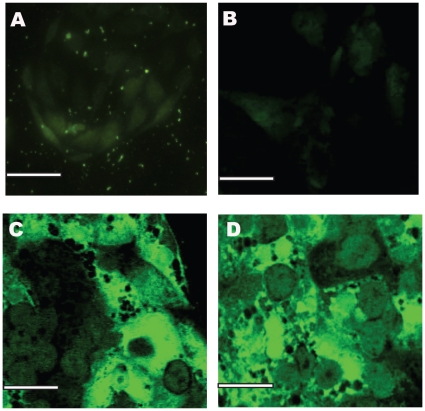
SE-1 immunostaining to monitor rLSEC phenotype, images obtained on day 12 in culture. **A**. rLSEC monolayer, **B**. 50K rLSEC-Hepatocyte, **C**. 50K rLSEC-5L-Hepatocyte, and **D**. 50K rLSEC-15L- Hepatocyte. Scale bar = 50 microns.

In LSECs, a cell-surface marker, recently identified as Cd32b, binds to the SE-1 antibody providing a convenient method to validate their phenotype [25], [39]. Immunostaining for the SE-1 antibody was conducted on days 4 and 12 for rLSECs cultured as a monolayer, with hepatocytes (rLSEC-Hepatocyte) and with PEM-coated hepatocytes (rLSEC-PEM-Hepatocyte). Four days after the isolation of rLSECs from the liver, rLSEC monolayers only exhibited weak fluorescence (**Figure 2A**) and rLSECs cultured with hepatocytes in the absence of a PEM appeared to have dedifferentiated (**Figure 2B**). However, 50K LSEC-5L-Hepatocyte and 50K LSEC-15L-Hepatocyte cultures maintained their phenotype, as indicated by green fluorescence (**Figure 2C and 2D**). By day 12, rLSEC monolayers did not exhibit any fluorescence. In contrast, rLSEC-PEM-Hepatocyte cultures exhibited bright green fluorescence (**Figure 3**). These results indicated that rLSECs maintained their phenotype in the 3D hepatic models.

**Figure 4:**
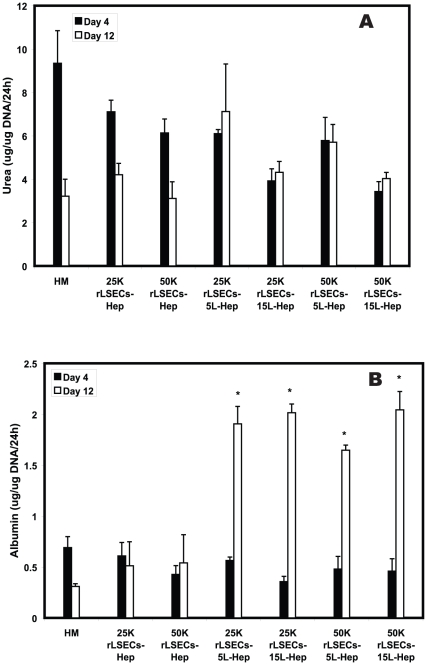
Urea secretion and albumin production over a 12 day culture period. **A**. Urea production **B**. Albumin secretion. An asterisk (*) indicates a statistically significant increase on day 12 in comparison to HMs. Hep = hepatocytes.

Urea and albumin production were monitored to determine whether the presence of rLSECs would stabilize or enhance hepatocyte function. These are two reliable markers of hepatocellular performance [12], [13]. Urea production monitored over a 12 day period indicated a decrease of 60%, 48% and 50% in hepatocyte monolayers (HMs), 25K rLSEC-Hepatocyte and 50K-rLSEC cultures respectively between day 4 and day 12 (**Figure 4A**) . In contrast, urea production either remained stable or increased in the rLSEC-PEM-Hepatocyte samples. For example, urea production increased approximately 18%, 10% and 20% in the 25K rLSEC-5L-Hepatocytes, 50K rLSEC-5L-Hepatocytes, and 50K rLSEC-15L-Hepatocytes, respectively.

Hepatocyte monolayers exhibited a decrease in albumin production by approximately 65% over the 12 day observation period (**Figure 4B**). 25K rLSEC-Hepatocyte samples exhibited a decrease of approximately 16% and 50K rLSEC-Hepatocyte cultures exhibited an increase of 28%. In contrast, all the 3D hepatic cultures exhibited a significant 3–6 fold elevation in albumin production. Specifically, the fold increases were 3.4, 5.7, 3.3, and 4.5 for 25K rLSEC-5L-Hepatocytes, 25K rLSEC-15L-Hepatocytes, 50K rLSEC-5L-Hepatocytes and 50K rLSEC-15L-Hepatocytes samples respectively. The increase in albumin secretion was statistically significant in each rLSEC-PEM-Hepatocyte cultures in comparison to hepatocyte monolayers with *p*-values lower than 0.05.

**Figure 5:**
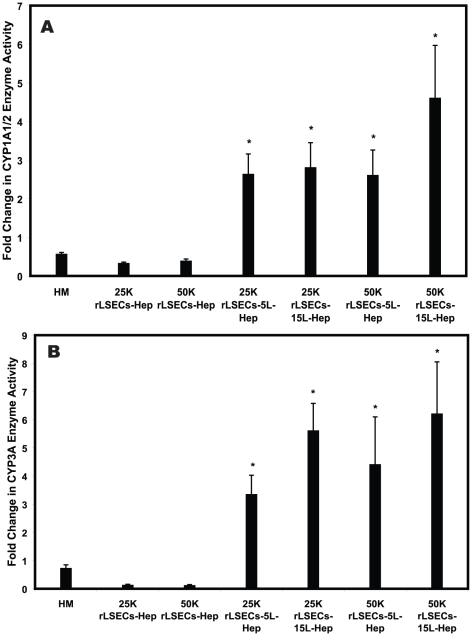
Fold change in CYP enzyme activity over a 12 day culture period. **A**. CYP1A1/2 **B**. CYP3A. An asterisk (*) indicates a statistically significant increase on day 12 in comparison to HMs. Hep = hepatocytes.

Cytochrome P450 enzymatic activity is another critical function of hepatocytes. This class of enzymes mediates the metabolism of multiple toxins, xenobiotics and pharmaceuticals [40]–[43]. Cytochrome P4501A1/2 (CYP1A1/2) and CYP3A activities were monitored in all cultures on days 4 and 12. Our goal was to determine if the enzymatic kinetics increased or decreased over the observation period, since decreasing enzymatic activity is usually exhibited due to deteriorating phenotype. Thus, we report the difference in CYP enzyme activity as a fold change between day 4 and day 12. CYP1A1/2 activity decreased by approximately 44% in hepatocyte monolayers and up to 67% in rLSEC-Hepatocyte cultures (**Figure** 5A). All rLSEC-PEM-Hepatocyte samples exhibited an increased CYP1A1/2 activity, with the fold increase ranging from 2.6 to 4.6. The greatest increase was observed in 50K rLSEC-15L-Hepatocyte samples. The increase in enzymatic activity in each rLSEC-PEM-Hepatocyte cultures was found to be statistically higher (*p*-value<0.05) in comparison to hepatocyte monolayers. A similar trend was observed for CYP3A enzymatic activity (**Figure** 5B). CYP3A enzymatic activity decreased by approximately 27%, 87%, and 87% in hepatocyte monolayers, 25K rLSEC-Hepatocyte and 50K rLSEC-Hepatocyte cultures, respectively. In contrast, CYP3A enzymatic activity increased in all rLSEC-PEM-Hepatocyte samples. The increase was 3–6 fold and was statistically significant in comparison to hepatocyte monolayers (*p*-value<0.05). Once again, the greatest increase was observed in 50K rLSEC-15L-Hepatocyte samples.

**Figure 6:**
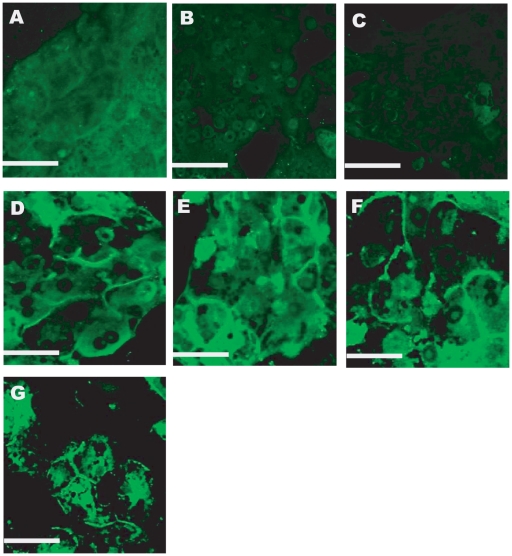
Dipeptidyl peptidase IV (DPP IV) immunostaining for bile canaliculi obtained on day 12 in culture. **A**. HM, **B**. 25K rLSEC-Hepatocyte, **C**. 50K rLSEC-Hepatocyte, **D**. 25K rLSEC-5L-Hepatocyte, **E**. 25K rLSEC-15L-Hepatocyte, **F**. 50K rLSEC-5L-Hepatocyte, **G**. 50K rLSEC-15L-Hepatocyte. Scale bar = 50 microns.

Immunostaining was conducted to determine the presence of bile canaliculi. These are channels through which bile acids are shuttled between the liver and intestines. Their presence indicates the polarization of hepatocytes [44], [45]. Hepatocyte monolayers and rLSEC-Hepatocyte cultures did not exhibit these channels on day 12 (**Figure** 6). In contrast, all four rLSEC-PEM-Hepatocyte samples exhibited well-defined canaliculi.

## Discussion

Due to the rapid dedifferentiation of hepatic parenchymal and non-parenchymal cells after they are isolated from the liver, the development of *in vitro* liver-like tissues has been non trivial. A liver model that recapitulates key features of hepatic sinusoids is a potentially powerful medium for obtaining comprehensive knowledge on metabolism, detoxification and signaling pathways. A critical component in the development of such a model is maintaining the phenotype of hepatic cells *in vitro*. As a first step towards establishing such a liver model, we have assembled 3D hepatic architectures comprised of primary hepatocytes, rLSECs and a polyelectrolyte-derived interface that acts as the Space of Disse. This model mimics the stratified structure of the liver *in vivo* and incorporates the two major cell types found in this organ.

Recent reports have emphasized the difficulty in maintaining LSEC phenotype beyond a few days in monolayer or co-cultures [24]–[26]. In the hepatic model reported herein, only rLSECs cultured in an rLSEC-PEM-Hepatocyte construct exhibited binding to the SE-1 antibody twelve days after isolation from the liver. Both monolayers of LSECs and cultures that did not contain a PEM exhibited a loss in phenotype after four days, a result that is in agreement with other studies [24], [25]. The physical properties of the PEM, specifically the modulus that closely mirrors bulk liver modulus, may have played a critical role in rLSEC adhesion. In addition, the high degree of hydration of the PEM may have potentially modulated inter-cellular signaling [30]. We hypothesize that cultures with lower concentrations of rLSECs (5000 or 10,000) did not maintain hepatocellular functions due to lower heterotypic cell-cell interactions. PEMs with greater than 15 layers also did not exhibit optimal function. One potential reason could be that the longer deposition process as well as the higher concentration of the polyelectrolytes could have resulted in imparting stress to hepatocytes.

A significant finding of our study was the three-six fold increase in albumin production in rLSEC-PEM-Hepatocyte cultures. These trends clearly indicate that hepatocellular functions are maintained as well, since albumin is one of the key markers of hepatic function. In contrast, previous reports on 2D co-cultures of rLSECs and hepatocytes, show that albumin secretion decreased as a function of time [25]. We hypothesize that that higher albumin secretion in our 3D models is due to significantly higher hepatocyte-rLSEC heterotypic interaction in comparison to conventional 2D co-cultures. These data further underscore the need for tissue engineered liver models that recapitulate the layered hepatic architecture found *in vivo*.

Optimal CYP enzymatic activity in hepatocytes is critical for the liver to conduct detoxification of a wide range of toxins, drugs and xenobiotics. Since LSECs are often the first cell type that comes in contact with a drug or toxin, models that do not include these cells will not provide relevant information required for therapeutic strategies of the future. A recent study showed that when a combination of alcohol and acetoaminophen was administered to the liver, significant rLSEC death and loss of actin cytoskeletal organization occurred [3]. A more significant outcome of this report was that LSECs were affected a few hours prior to any effect on hepatocytes. Thus, *in vitro* cultures that do not include LSECs cannot capture the unique inter-cellular mechanisms that govern detoxification and the subsequent effect on other liver metabolites.

We focused our attention on two major classes of CYP enzymes, CYP1A1/2 and CYP3A, since these enzymes typically mediate the metabolism of polyaromatic compounds and pharmaceuticals. Once again, the rLSEC-PEM-Hepatocyte cultures exhibited increasing enzymatic activity over the twelve day observation period. These trends were mirrored in the immunostaining experiments for bile canaliculi. Multiple cytochrome P450 enzymes are often involved in the biotransformation of bile acids [45]–[47]. Furthermore, bile canaliculi are the channels through which metabolites of the detoxification processes are transported away from the liver. Therefore, together these data suggest that rLSEC-PEM-Hepatocyte cultures are highly suitable models to monitor the effects of drugs and toxins on the liver.

Complications arising from drug-drug and drug-toxin interactions are becoming an increasing concern for human health. There is a need to develop liver-like tissue models that can serve as accurate models for drug and toxicity testing. Such models cannot rely on the use of a single hepatic cell type. Instead, the inclusion of multiple cell types found in the liver is a pre-requisite to obtaining a comprehensive overview on all perturbed liver functions. In the current study, the inclusion of hepatocytes and rLSECs in a layered 3D model resulted not only in the simultaneous maintenance of their individual phenotype but also in increased hepatocellular functions. The intricate inter-cellular signaling pathways through which these cells communicate within the 3D liver model will be the focus of future studies.
